# Identification of genes involved in the tomato root response to *Globodera rostochiensis* parasitism under varied light conditions

**DOI:** 10.1007/s13353-024-00897-6

**Published:** 2024-08-14

**Authors:** Mateusz Matuszkiewicz, Magdalena Święcicka, Marek D. Koter, Marcin Filipecki

**Affiliations:** Department of Plant Genetics, Breeding and Biotechnology, Institute of Biology, Warsaw University of Life Sciences - SGGW, Nowoursynowska 159, Warsaw, 02-776 Poland

**Keywords:** Cyst nematodes, Stress combination, Abiotic stress, Biotic stress, RNA-seq

## Abstract

Understanding the intricate interplay between abiotic and biotic stresses is crucial for deciphering plant responses and developing resilient cultivars. Here, we investigate the combined effects of elevated light intensity and nematode infection on tomato seedlings. Chlorophyll fluorescence analysis revealed significant enhancements in PSII quantum yield and photochemical fluorescence quenching under high light conditions. qRT-PCR analysis of stress-related marker genes exhibited differential expression patterns in leaves and roots, indicating robust defense and antioxidant responses. Despite root protection from light, roots showed significant molecular changes, including downregulation of genes associated with oxidative stress and upregulation of genes involved in signaling pathways. Transcriptome analysis uncovered extensive gene expression alterations, with light exerting a dominant influence. Notably, light and nematode response synergistically induced more differentially expressed genes than individual stimuli. Functional categorization of differentially expressed genes upon double stimuli highlighted enrichment in metabolic pathways, biosynthesis of secondary metabolites, and amino acid metabolism, whereas the importance of specific pathogenesis-related pathways decreased. Overall, our study elucidates complex plant responses to combined stresses, emphasizing the importance of integrated approaches for developing stress-resilient crops in the face of changing environmental conditions.

**Supplementary Information:**

The online version contains supplementary material available at 10.1007/s13353-024-00897-6.

## Introduction

Plants have evolved to thrive in environmental conditions which vary within the range of variability characteristic of a given climatic zone and ecosystem. Moreover, they try to endure more or less regular pression of biotic threats such as diseases and pests. On top of this, there is a pressure of climate changes including recent anthropogenic global warming and environment pollution. All of these factors, usually acting simultaneously, affect crop plant growth development and yield making the study of adaptation mechanisms increasingly important (Zandalinas and Mittler 2022). The ability to detect and adjust reactions applies to both abiotic (drought, salinity, heat, cold, chilling, freezing, nutrient deficiency, varying light intensities, UV radiation, ozone exposure, and anaerobic conditions caused by flooding) and biotic stimuli (bacteria, fungi, viruses, oomycetes, and a multitude of herbivorous animals) (Zandalinas et al. 2023). Hans Selye (1936) postulated conception of stress response for living organisms, which can be synthetized to: “All agents can serve as stressors, inducing both stress and specific actions. These caused coexistence of stressor-specific and general non-specific responses.” “True stress” occurred when a specific threshold of a stressor surpasses the compensatory capacity of the plant. The stress tolerance threshold is contingent not solely upon the plant species but also on the nature of applied stressors and the conditions of the plant. Additionally, variations exist among plants in terms of their capacity to cope with stress. This means that stress is a dose-dependent and species-specific type of plant response (Lichtenthaler 1998; Georgieva and Vassileva 2023). Consequently, plants constantly confront diverse combinations of several stimuli or stressors (Anwar et al. 2021). Zandalinas and colleagues (2021) introduced the term “multifactorial stress combination” (MFSC) to characterize situations where three or more stress-inducing factors co-occur. Even if the number of MFSC is very high, the number of physiological and molecular responses tends to integrate towards relatively limited repertoire of adaptive or defense mechanisms. Consequently, the individual impact of a given stressor may be different than their collective effect showing synergy or antagonism. Importantly the molecular outcome of underlaying mechanisms is distinctly discernible at the level of the plant’s transcriptome, proteome, or metabolome being the source of potential targets to enhance crop performance via, e.g., genome editing (Zandalinas et al. 2023).

Most of studies with combined stress conditions indicate that there is a negative impact of abiotic stress (mainly drought and salinity stress) on pathogen resistance (Suzuki et al. 2014). There are however some exceptions such as the beneficial role of salt-related stress on the powdery mildew resistance of barley (Wiese et al. 2004). Such unpredictable reaction may depend on the dual role of reactive oxygen species (ROS) and the specific and local interplay of ROS stoichiometry and pathogen effectors (Gadjev et al. 2006; Siddique et al. 2014).

Plant parasitic nematodes are among the most significant pests, with those belonging to the genera *Globodera* and *Heterodera*, known as cyst-forming nematodes, ranking at the top of the list, and causing annual losses ranging from US$ 80 to 358 billion (Jones et al. 2013). These nematodes infect roots, suppressing the defense response and inducing plant cells to form syncytia specialized in sustaining the nutrition of developing larvae. During syncytium formation, numerous morphological, ultrastructural, physiological, and molecular changes occur in the infected plant. Particularly, the initial syncytial cell undergoes significant enlargement, the nucleus swells, and the central vacuole is replaced by smaller ones. Mitochondria, plastids, endoplasmic reticulum, lipid bodies, and ribosomes proliferate, causing the cytoplasm to become denser. The syncytium gradually enlarges through partial dissolution of cell walls and fusion of neighboring protoplasts (Matuszkiewicz and Sobczak 2024). All these changes are accompanied by local or systemic alterations in the expression of genes related to various processes such as the cell cycle, cell wall modification, ROS homeostasis, and defense. Additionally, there is a shift in the expression of genes involved in signal transduction and hormonal regulation (Matuszkiewicz et al. 2018; Siddique et al. 2022).

Despite advancements in understanding plant responses to various stimuli, there remain a limited number of studies examining the concurrent effects of biotic or abiotic stressors alongside plant-parasitic nematodes. Kutyniok et al. (2014a) conducted a study investigating the impact of nematode (*H. schachtii*) parasitism on *Arabidopsis* plants concurrently attacked by aphids (*Myzus persicae*). Their findings indicated that the sequence of parasite feeding on a host plant reciprocally affected their fitness and reproductive success. Notably, the effect of beet cyst nematode on aphid performance was found to be modified by nitrate fertilization (Kutyniok and Müller 2013). Additionally, the researchers presented microarray transcriptome data illustrating that aphid infestation influenced nematode-induced transcription in roots, but not vice versa (Kutyniok et al. 2014b). Kammerhofer et al. (2015) further explored the communication between roots and leaves subjected to biotic stimuli. They simultaneously infested *Arabidopsis* roots with *H. schachtii* and leaves with *Frankliniella occidentalis* or *Tetranychus urticae*. The study revealed that *H. schachtii* triggered hormone-related systemic responses, leading to elevated levels of jasmonic acid (JA), salicylic acid (SA), and indoleacetic acid (IAA) in aboveground parts of plants. These findings elucidated the altered susceptibility and/or attraction of shoot invaders to these plants. Interestingly, while shoot-feeding herbivores (thrips and spider mites) modified *Arabidopsis* phytohormone homeostasis, only *F. occidentalis* increased susceptibility to *H. schachtii* by altering JA and its active conjugate JA-Ile in the roots. Several studies have also investigated the combined effects of abiotic stress and nematode parasitism. For example, in-depth physiological studies on upland rice led to the conclusion that the presence of plant-parasitic nematodes exacerbates the detrimental effects of drought (Audebert et al. 2000). Yang et al. (2015) discovered that nighttime irradiation of tomato and watermelon plants with different light qualities influenced susceptibility to the root knot nematode — *Meloidogyne incognita*. Notably, treatment with red light significantly boosted immunity, correlating with increased expressions of PR1 (a marker gene of SA signaling activation) and PI1 (a marker gene of JA signaling). Moreover, this treatment improved plant growth and leaf CO_2_ assimilation. The molecular investigation of the combination of drought stress and *H. schachtii* infection was conducted by Atkinson et al. (2013) in *Arabidopsis* roots. Detailed transcriptome analysis showed that 47% of genes expressed differentially during drought stress, and 85% of the genes involved in the response to *H. schachtii* parasitism did not undergo differential expression under cumulative stress. Further studies focused on selected genes — *RAPID ALKALINIZATION FACTOR-LIKE8* (*AtRALFL8*), *METHIONINE GAMMA LYASE* (*AtMGL*), and *AZELAIC ACID INDUCED1* (*AtAZI1*) — highlighting the intricate interplay among various stress responses in plants, affirming the significance of investigating combined stress factors.

Similar to other abiotic stresses, substantial changes in light intensity and quality have negative effects on plant growth due to the deregulation or damage of photosynthetic machinery by excess energy. Plants have therefore evolved various protective and compensating mechanisms that monitor the intensity, wavelength, duration, and direction of light, and respond to mitigate the negative effects of harsh conditions (Roeber et al. 2021). Chloroplasts seem to play a central role not only in sensing and responding to environmental stresses but also in orchestrating immune reactions against plant pathogens. They are well-adapted for this role as they are the primary source of ROS and the starting point of SA biosynthesis in plants (Trotta et al. 2014; Lefevere et al. 2020). Consistently, light stress induces systemic acclimatization, enhancing tolerance to virulent bacteria through local and systemic changes in the pool of ROS, SA, and ethylene (Szechyńska-Hebda et al. 2010). The data mentioned clearly indicate numerous points of intersection in the responses to biotic stress and light stress.

We conducted this study to enhance the understanding of how different light intensities influence the molecular response of tomato (*Solanum lycopersicum*) roots to parasitism by the potato cyst nematode (*Globodera rostochiensis*).

## Materials and methods

### Plant material

Seeds of tomato (*Solanum lycopersicum*) cv. Moneymaker were used in experiments. Seeds were surface-sterilized in 1.5% sodium hypochlorite for 10 min and subsequently rinsed three times in distilled water. Two seeds per Petri dish (100 mm in diameter) were sowed on medium containing 1.5% (w/v) B5 medium (Gamborg’s basal salt mixture, 2% (w/v) sucrose, and 1.5% (w/v) agar, pH 6.2) and subsequently grown at a long day regime (16:8 h light/dark, 22:20 °C, 70% HR). Light intensity was between 50–60 μmol m^−2^ s^−1^ — low light conditions (LL) — and 350–400 μmol m^−2^ s^−1^ — high-light conditions (double stimuli; HL). Petri dishes were sealed with gas permeable medical adhesive tape (3 M™ Micropore™). To avoid root system illumination, Petri dishes were covered in black envelopes.

### Nematode assay

Cysts of potato cyst nematode (*G. rostochiensis* Woll.) pathotype Ro1 were surface sterilized in 90% (v/v) ethanol for 15 s following a 10-min incubation in 1.3% (w/v) sodium hypochlorite. Then, cysts were washed 3 times in sterile water, and rehydrated in sterile potato root diffusates in the dark at 20 °C for 1 week. The potato root diffusate was made according to method established by Evans (1983). Hatched pre-parasitic J2s were sterilized by 0.05% HgCl_2_ for 5 min and immediately washed five times in distilled water. After sterilization suspended in sterile distilled water nematodes were checked for their vitality and density.

Fourteen-day-old tomato seedlings were inoculated with 200–250 J2s under sterile conditions. Inoculated plates were kept in the dark for 6 h, and subsequently transferred into a growth chamber under high-light conditions. Two plants were used in one Petri dish and the experiments were repeated three times with 10 plants per genotype in one replicate. The numbers of induced syncytia per root system were counted at 14 days post inoculation (dpi) and the data were analyzed by a *t*-test (*p* < 0.05).

### Chlorophyll a fluorescence measurement

Chlorophyll a fluorescence was determined using a pulse amplitude-modulated FluorCam 800 MF PSI device (Brno, Czech Republic) on whole *S. lycopersicum* leaves. Before taking measurements, the plants underwent a 30-min period of dark adaptation to determine the initial fluorescence (*F*_o_) and the maximum fluorescence (*F*_m_). According to the methodology established by Baker (2008), the maximum quantum efficiency of PSII — *F*_v_/*F*_m_ = (*F*_m_ − *F*_o_)/*F*_m_; non-photochemical quenching — NPQ = (*F*_m_ − *F*_mʹ_)/F_mʹ_; photochemical quenching — qp = (*F*_mʹ_ − *F*_t_)/(*F*_mʹ_–*F*_0ʹ_); and the operating quantum efficiency of PSII known as PSII quantum yield — ΦPSII = (*F*_mʹ_ − *F*_s_)/*F*_mʹ_ were calculated. The plant vitality index *R*_fd_ was calculated by the FluorCam 7.0 software. Data were further statistically analyzed using two-way ANOVA with Bonferroni test for correction for multiple comparisons (*p* < 0.05).

### RNA extraction for transcriptomic analysis

Total RNA was isolated from uninfected plants (both leaves and roots) at 1st and 3rd days post transfer (dpt), and infected root segments, along with appropriate controls, were collected 14 dpi. The isolation was performed using the Universal RNA Purification Kit (Eurx, Gdansk, Poland) following the manufacturer’s protocol, which included on-column digestion of DNA. RNA integrity was evaluated on 1% agarose gel. However, RNA yield and purity were estimated using the NanoDrop ND-1000 (NanoDrop Products, Wilmington, DE, USA), and the Experion (Bio-Rad, Hercules, CA, USA). Total RNA with RQI values ≥ 9.0 and 28S:18S ratios ≥ 1.2 was used in the RNA-sequencing analysis.

### RNA-sequencing analysis

The Illumina HiSeq2500 platform (Illumina Inc., San Diego, CA, USA) was used for RNA-sequencing (RNA-seq) analysis. To obtain a comprehensive overview of the tomato root transcriptome and transcript profiles in response to *G. rostochiensis* parasitism under increased light intensity, three biological replicates were used to construct the libraries. A total of 18 cDNA libraries were sequenced by Genomed SA (Warsaw, Poland) with paired-end sequencing (Supplementary Fig. 1).

In all analytical procedures, the ITAG4.1 Tomato Genome Annotation Release file, obtained from Solgenomics (https://solgenomics.net), was employed. Initially, the ITAG4.1_gene_models.gff file was subjected to conversion into the ITAG4.1_gene_models.gtf file format, utilizing the gffread software version 0.11.7 (Pertea and Pertea 2020). The quality assessment and trimming of fastq files were executed with Trim Galore version 0.6.4 (Krueger et al. 2023). Subsequently, the STAR aligner version 2.7.3a (Dobin et al. 2013) was utilized to index the genome and align reads to the tomato genome assembly build 4.00. Mapped reads or fragments, in the case of paired-end data, were associated with genomic features, generating bam files, through the featureCounts function from the Rsubread package version 2.14.2 (Liao et al. 2019), integrated into R software version 4.3.0. The resulting count matrix was then subjected to the identification of differentially expressed genes (DEGs) employing the DESeq2 package version 1.40.2 (Love et al. 2014) within R software version 4.3.0. The criteria for DEG selection were set at |log2-fold change (FC)|> 1.0 and adjusted P-value < 0.05. Finally, DEGs were annotated by referencing the ITAG4.1_descriptions.txt file from Solgenomics (https://solgenomics.net) with the aid of the dplyr package version 1.1.3 (Wickham et al. 2023) within R software version 4.3.0. The software tools, namely gffread, Trim Galore, and STAR, were executed on an operating system environment running Ubuntu 20.04.5 LTS (GNU/Linux 4.4.0–19041-Microsoft x86_64). Gene ontology enrichment analysis was performed with ShinyGO v.0.77 (Ge et al. 2020) The sequencing data are accessible in SRA database (PRJNA1078223).

### Quantitative real-time RT-PCR (qRT-PCR)

The pooled plant materials, obtained from three individual plants were used as a singular biological replication. In all qRT-PCR experiments, we used three biological replicates. RNA was isolated using the Universal RNA Purification Kit (Eurx, Gdansk, Poland) according to the manufacturer’s protocol with on-column digestion of DNA. RNA integrity was assessed using a 1% agarose gel, while RNA yield and purity were determined using the NanoDrop ND-1000 (NanoDrop Products, Wilmington, DE, USA). A total of 1 µg of RNA was reverse transcribed using (N) 6 random hexamer primers and following the conditions specified in the *QuantiTect* Reverse Transcription Kit (Qiagen). Quantitative RT-PCR was performed in triplicate using the *QuantiTect* SYBR Green PCR Kit (Qiagen) with the Bio-Rad CFX96 Touch™ Real-Time PCR Detection System (Bio-Rad, Hercules, CA, USA). The reaction conditions were as follows: denaturation at 95 °C for 3 min, and 40 cycles of 95 °C for 10 s and 60 °C for 30 s. The reaction mixture, with a total volume of 20 µL, consisted of 8 μL of cDNA (2.5 ng/μL), 1 μL for each gene-specific primer (10 mM), and 10 μL of the 2 × Ready Fast Green Mix reagent (from Biochem Development, Gdansk, Poland). Two tomato genes, SAND (SGN-U316474) and RPL8 (NM_001247186), were used as internal reference genes. The transcript level of the selected genes was normalized to that of SAND and RPL8 using the ^ΔΔ^Ct method (Livak and Schmittgen 2001). The significance of differences from the control was revealed by REST (Pfaffl et al. 2002). After the PCR, product melting curves were generated to verify the purity of the amplicons. The same methodology as described above was used for validation of the RNA-seq data (Supplementary Table 1). The list of primers used in qRT-PCR is included in the supplementary materials (Supplementary Table 2).

## Results

### Effects of elevated light intensities on photosynthetic performance and molecular response in tomato seedlings

**Fig. 1:**
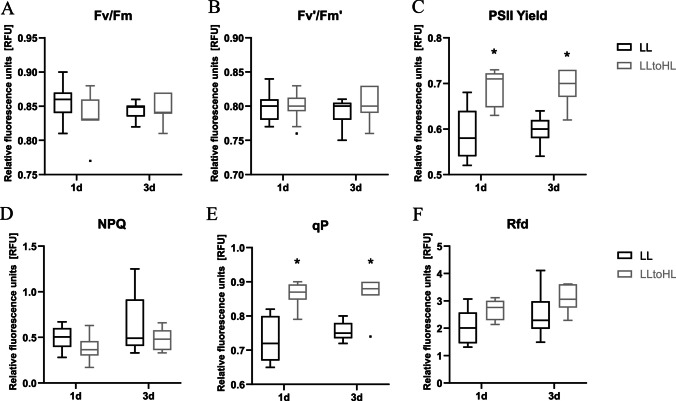
Chlorophyll a fluorescence in tomato seedlings after transferring to elevated light intensities. (A) Maximum quantum efficiency of PSII photochemistry (*F*_v_/*F*_m_); (B) PSII quantum yield in light-adapted leaves (*F*_v′_/*F*_m′_); (C) PSII quantum yield (PSII Yield); (D) non-photochemical quenching (NPQ); (E) photochemical fluorescence quenching (qP); (F) plant vitality (*R*_fd_). Distribution of data was presented in boxplots (median, quartiles, and potential outliers) from three independent experiments, each containing 3–5 plants per treatment. Statistical analysis was performed by using two-way analysis of variance (ANOVA). Bonferroni test was used for correction for multiple comparisons

Commonly employed non-invasive assessments of a plant’s physiological state rely on measuring chlorophyll a fluorescence. In this study, we aimed to investigate the combined reaction to high light intensity and nematode infection; therefore, the conditions typically used in tomato/potato cyst nematode experiments were slightly modified (Dąbrowska-Bronk et al. 2015; Święcicka et al. 2017). Tomato seedlings were exposed to elevated light intensities being placed on a medium in a plastic Petri dish sealed with permeable medical adhesive tape. The shoots remained uncut, while the roots were shielded with a black envelope. The maximum light intensity was adjusted to a level that did not cause temperature shifts. The impact of transferring tomato plants from low light (LL) to high light (HL) conditions on photosystem II (PSII) photochemistry was assessed using various chlorophyll fluorescence–related parameters, including maximum quantum efficiency of PSII (*F*_v_/*F*_m_), non-photochemical quenching (NPQ), photochemical fluorescence quenching (qP), PSII quantum yield (ΦPSII), PSII quantum yield in light-adapted leaves (*F*_v_′/*F*_m_′), and plant vitality (*R*_fd_) (Baker 2008). Measurements were taken at two time points: 1 and 3 dpt, with appropriate LL controls on 2-week-old tomato seedlings. Two parameters exhibited statistically significant increases after transferring plants to elevated light conditions at both time points: PSII quantum yield and photochemical fluorescence quenching (Fig. 1 C and E). The other parameters showed insignificant fluctuations (Fig. 1 A, B, D, and F). These results indicate that seedlings cultured under higher light intensities had greater efficiency of PSII, consistent with previous literature (Takagi et al. 2019).

To track the activation of signaling pathways, we evaluated the expression of several stress-related marker genes using quantitative reverse transcription-polymerase chain reaction (qRT-PCR) analysis. The expression was checked in leaves (organs subjected to HL stimuli) and roots (remote, shaded organs) at 1 and 3 dpt. It is worth noting that the selected markers represent not only general stress or light response pathways but are also important in specific nematode responses, especially during the first days of infection, as described in the literature (Huang et al. 2019; Matuszkiewicz and Sobczak 2024).

**Fig. 2:**
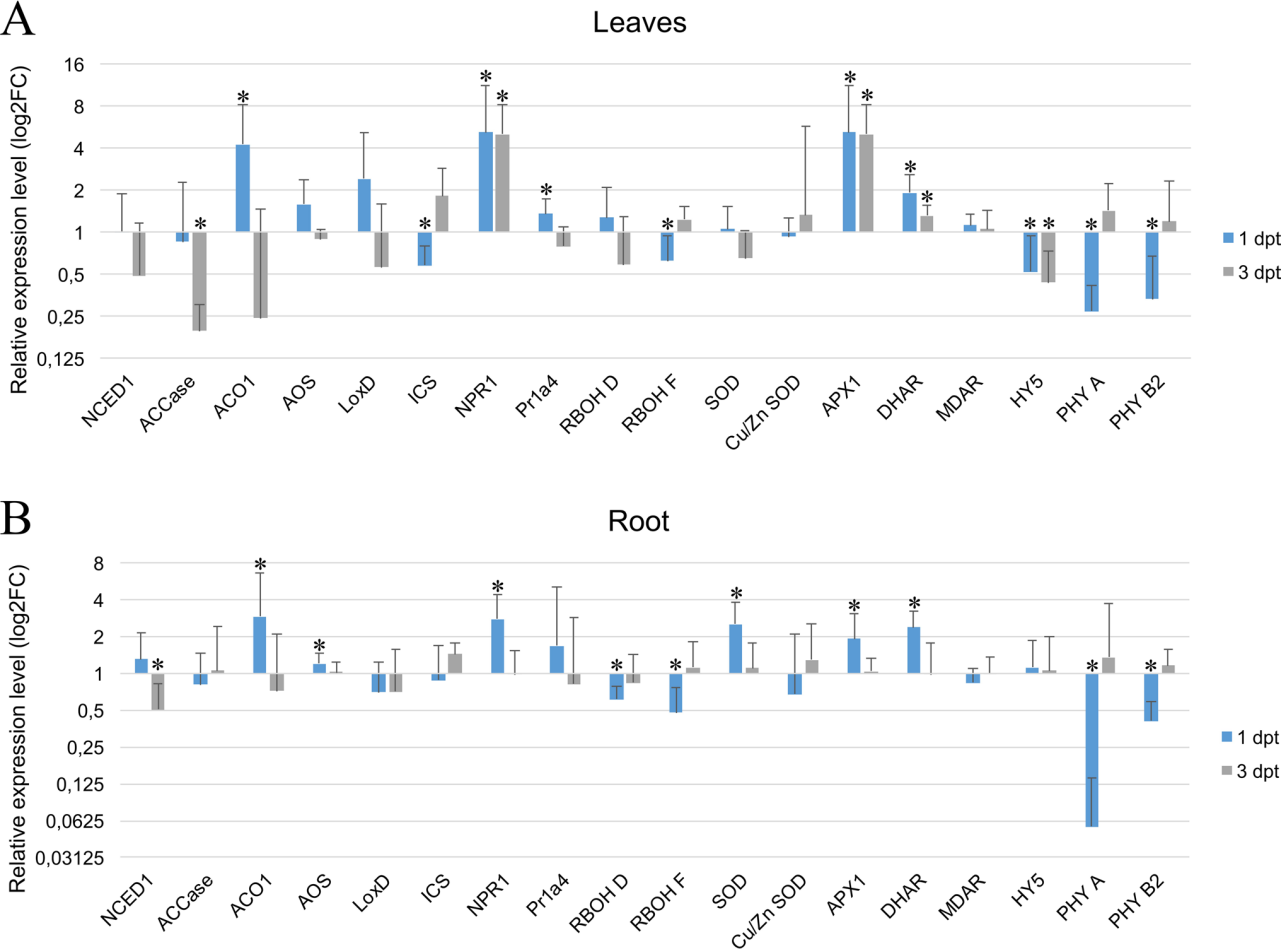
Gene expression analysis in leaves and roots of tomato plants. qRT-PCR analysis of marker genes linked to photochromes, ROS metabolism, and phytohormones related to biotic stress were examined in leaves (**A**) and roots (**B**) of tomato plants after transferring to elevated light intensities. The expression levels of target genes were quantified with reference to the expression of *RPL8* and *SAND* compared to the control (plants grown in low light intensities). The relative expression levels are shown as logarithm of fold changes relative to the copy number of a particular mRNA gene in the control sample. Results are the means (± SEM; standard error of mean) from three independent experiments. The asterisks indicate the significant differences from the control as revealed by REST (Pfaffl et al. 2002) (*p* < 0.05)

In leaves, the strongest upregulation at 1 dpt was observed in the *ACO1*, *NPR1*, *Pr1a4*, *APX1*, and *DHAR* genes, while downregulation was observed for *ISC*, *HY5*, *PHYA*, and *PHYB2* transcripts (Fig. 2A). Changes in gene expression at 3 dpt were less widespread, with upregulation of *NPR1*, *APX1*, and *DHAR* and downregulation of *ACCase* and *HY5*. The observed changes in leaves may be attributed to the activation of defense and antioxidant pathways to counteract oxidative stress and damage caused by excessive light. Simultaneously, downregulation of *ACCase*, *ICS*, and *HY5* may aim to conserve energy, possibly due to *ACCase*’s role in lipid metabolism and fatty acid synthesis, while also reducing susceptibility to light-induced stress.

Interestingly, the tomato root system was more sensitive to applied conditions, even when protected from light. We observed downregulation of *RBOHD* and *RBOHF* and upregulation of *APX1*, *SOD*, and *DHAR* among oxidative stress–sensitive genes. Genes involved in SA, JA, and ET signaling were upregulated. Notably, both *PHYA* and *PHYB2* were strongly downregulated in roots (Fig. 2 B). At 1 dpt, all the mentioned gene expression changes were observed, while at 3 dpt, only *NCED1* (involved in ABA biosynthesis) exhibited statistically significant alterations in roots, suggesting effective adaptation to unfavorable conditions and moderation of stress-related responses.

### Higher light intensities modify PSII photochemistry during *G. rostochiensis* parasitism

**Fig. 3:**
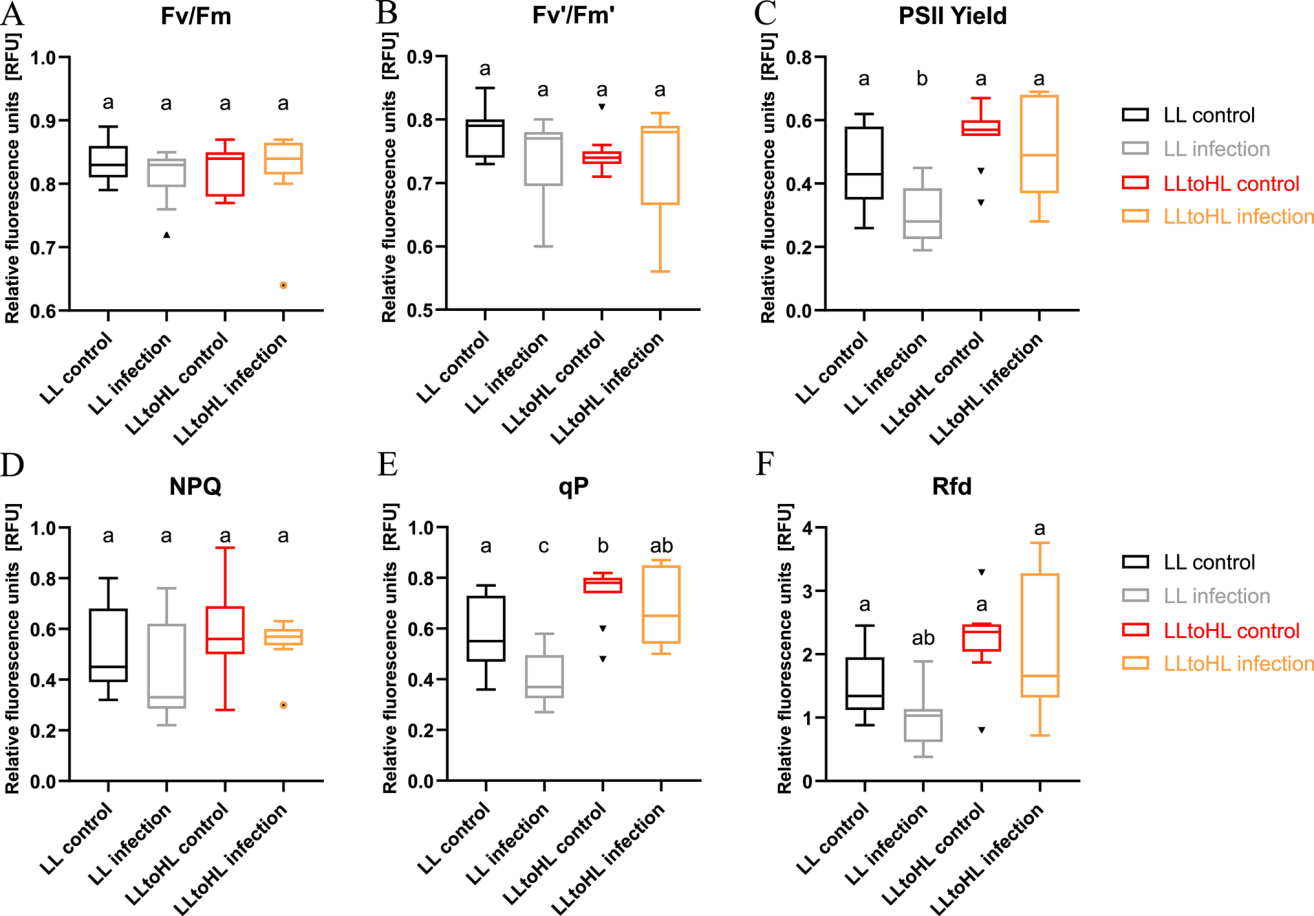
Chlorophyll a fluorescence in infected tomato seedlings after transferring to elevated light intensities. (A) Maximum quantum efficiency of PSII photochemistry (*F*_v_/*F*_m_); (B) PSII quantum yield in light-adapted leaves (*F*_v_′/*F*_m_′); (C) PSII quantum yield (PSII yield); (D) non-photochemical quenching (NPQ); (E) photochemical fluorescence quenching (qP); (F) plant vitality (*R*_fd_). Distribution of data was presented in boxplots (median, quartiles, and potential outliers) from three independent experiments, each containing 3–5 plants per treatment. Statistical analysis was performed by using one-way analysis of variance (ANOVA). Tukey test was used for correction for multiple comparisons

The introduction of a second stress factor can significantly alter the plant’s response across various dimensions, including physiological, biochemical, and molecular aspects. Therefore, our objective was to investigate how changes in light conditions might impact the susceptibility of tomatoes and whether these alterations would affect the efficiency of the photosynthetic apparatus. Among the six parameters measured, three showed differences associated with the combination of nematode parasitism and the transfer of plants to different light regimes: PSII yield, qP, and *R*_fd_. In plants infected with *G. rostochiensis* and cultivated in low light (LL) conditions, the PSII yield decreased. However, when infected plants were transferred to high light (HL) conditions, their PSII yield levels were similar to those of both control groups of plants. A similar trend was observed for photochemical fluorescence quenching and the parameter representing plant vitality. Infected plants acclimated to LL conditions exhibited a photoinhibition-like response, as evidenced by a decrease in the aforementioned parameters (see Fig. 3).

### Higher light intensities do not affect number of *G. rostochiensis* developing on tomato roots

**Fig. 4:**
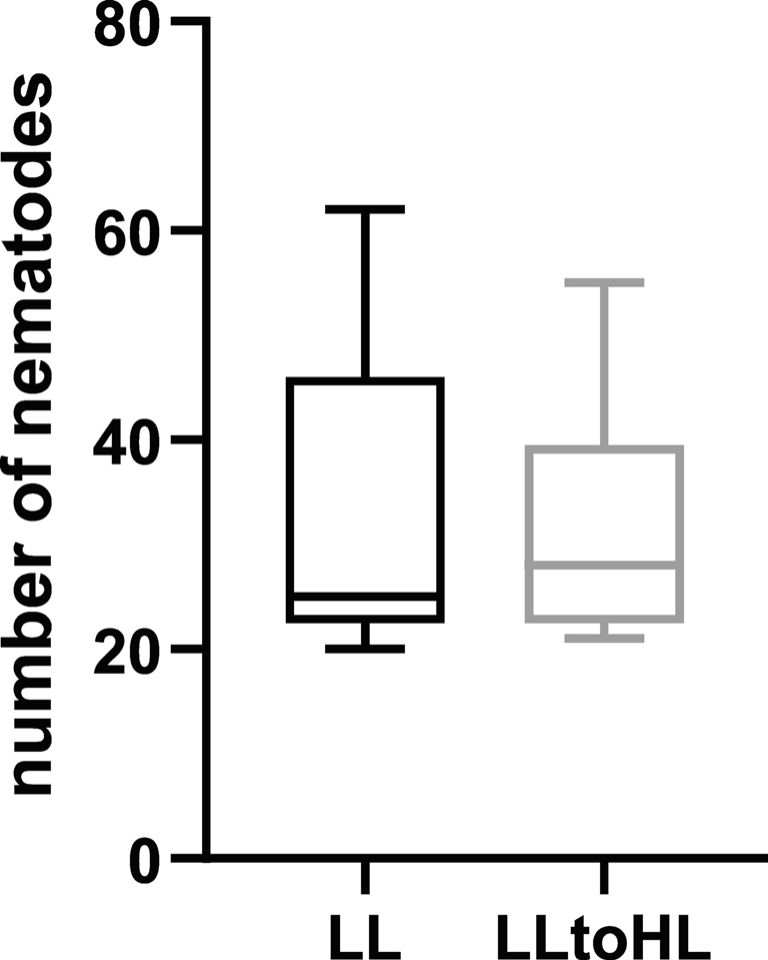
Nematode infection assay on tomato roots infected by *Globodera rostochiensis* pathotype Ro1. The numbers of developed syncytia were counted at 14 dpi and represented as boxplots. Data was collected from three independent experiments. Statistical analysis was performed by using *t*-test (*p* < 0.05)

The interplay between plant roots and cyst nematodes is a highly complex and extended process involving developmental and metabolic changes of plant cells, along with responses to damages, molecular patterns, and effectors associated with parasite activity (Matuszkiewicz and Sobczak 2024). The observed changes in photosynthetic efficiency and gene expression in non-infected plants may translate into the susceptibility level of the tomato (see Figs. 1 and 2). To test this hypothesis, we quantified the number of induced syncytia on tomato root systems in plants that were either continuously grown in low light (LL) conditions or transferred to high light (HL) intensities, and we did not observe any differences (see Fig. 4). It is worth mentioning that we did not observe changes in the morphology of the root system in both comparisons, which could potentially interfere with the level of susceptibility.

### Transcriptome analysis reveals DEGs in response to different light conditions during *G. rostochiensis* parasitism in tomato

Transcriptome profiling has become the fundamental diagnostic tool for monitoring plant reactions to stress, with RNA-seq being increasingly favored over microarrays, systematic RT-qPCR, SAGE, or cDNA-AFLP due to its advantages. Despite the importance of the tomato/PCN interaction, there is still a lack of RNA-seq perspective. In this study, we employed RNA-seq to investigate the regulatory networks active in nematode-infected roots subject to varying light conditions. Our investigation involved analyzing RNA-seq data derived from plants persistently grown under both low light (LL) and high light (HL) conditions, as well as plants subjected to a transition from LL to HL intensities. This comparative analysis aimed to elucidate the complexity of the response to dual stimuli. We also included non-infected plants subjected to an increase in light intensity, characterizing this scenario as a “light response.”

**Fig. 5:**
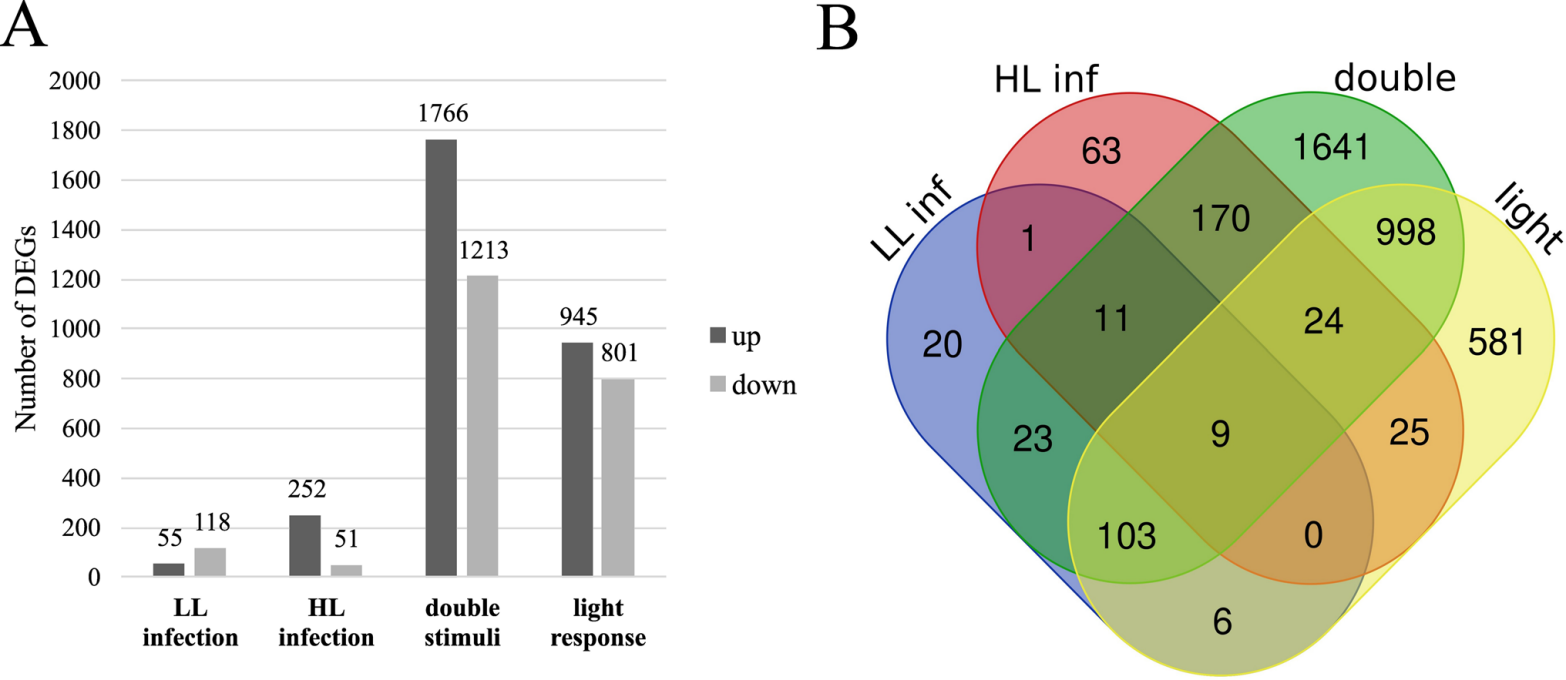
Number of DEGs after infections with *G.rostochiensis* at 14 dpi in roots containing syncytia. (A) Total number of DEGs in response to different conditions. (B) Venn diagram presenting the DEGs grouped according to the changes in their expression relative to the type of comparison

An initial noteworthy observation was that, despite the tomato susceptibility to *G. rostochiensis* not changing across distinct light conditions, we detected substantial alterations in the abundance of differentially expressed transcripts. The smallest number of DEGs, 173, was observed when comparing transcriptomes of infected LL-grown roots to control samples. In HL conditions, this comparison yielded nearly twice as many DEGs (303). The highest number of DEGs emerged in the double stimuli comparison — 2979 — while the light response alone resulted in 1746 DEGs (Fig. 5A). In all comparisons, upregulated DEGs constituted the predominant group, with the exception of the LL comparison, where downregulated genes accounted for 68% of the total DEGs (Fig. 5A). This indicates that, despite relatively small differences observed in DEGs between LL and HL conditions, light exerts a dominant influence on the regulation of gene expression. Moreover, light and nematode response synergistically interact, yielding more DEGs than the sum of individual stimuli.

The overlap of the aforementioned groups of DEGs would indicate more general mechanisms of plant reaction to biotic and abiotic stimuli (Fig. 5B). For example, in the group common for all four comparisons (9 DEGs), we found genes such as Zinc finger protein (*Solyc08g006470.5*), Peroxidase (*Solyc10g078890.2*), Glutathione S-transferase (*Solyc09g011540.2*), and Defensin protein (*Solyc07g007750.3*). Interestingly, in the common pool of DEGs for nematode-related response (11 DEGs), genes involved in secondary metabolism such as O-methyltransferase (*Solyc06g064510.2*), Flavin-containing monooxygenase (*Solyc08g068160.2*), Glycosyltransferase (*Solyc11g007460.1*), 2-oxoglutarate (*Solyc11g072110.2*), and ABA 8′-hydroxylase (*Solyc04g078900.3*) were present. Additionally, the analysis of DEGs revealed transcripts unique for each treatment, indicating specific processes related to the tested variables and their synergy. The smallest number of exclusive DEGs was found in the LL comparison, while the highest number was observed under double stress conditions, totaling 1641 DEGs (Fig. 5B). Here, we found genes involved in defense response, hormone homeostasis, ROS signaling, and regulation of primary and secondary metabolic processes (Supplementary Table 3).

Our analysis of DEGs uncovered a remarkable dynamic range of FC-value for several genes throughout the comparisons. However, as usual, the highest changes were detected for genes with very low expression. For a more detailed description, see Supplementary Table 3.

### Categorization of differentially expressed genes

**Fig. 6:**
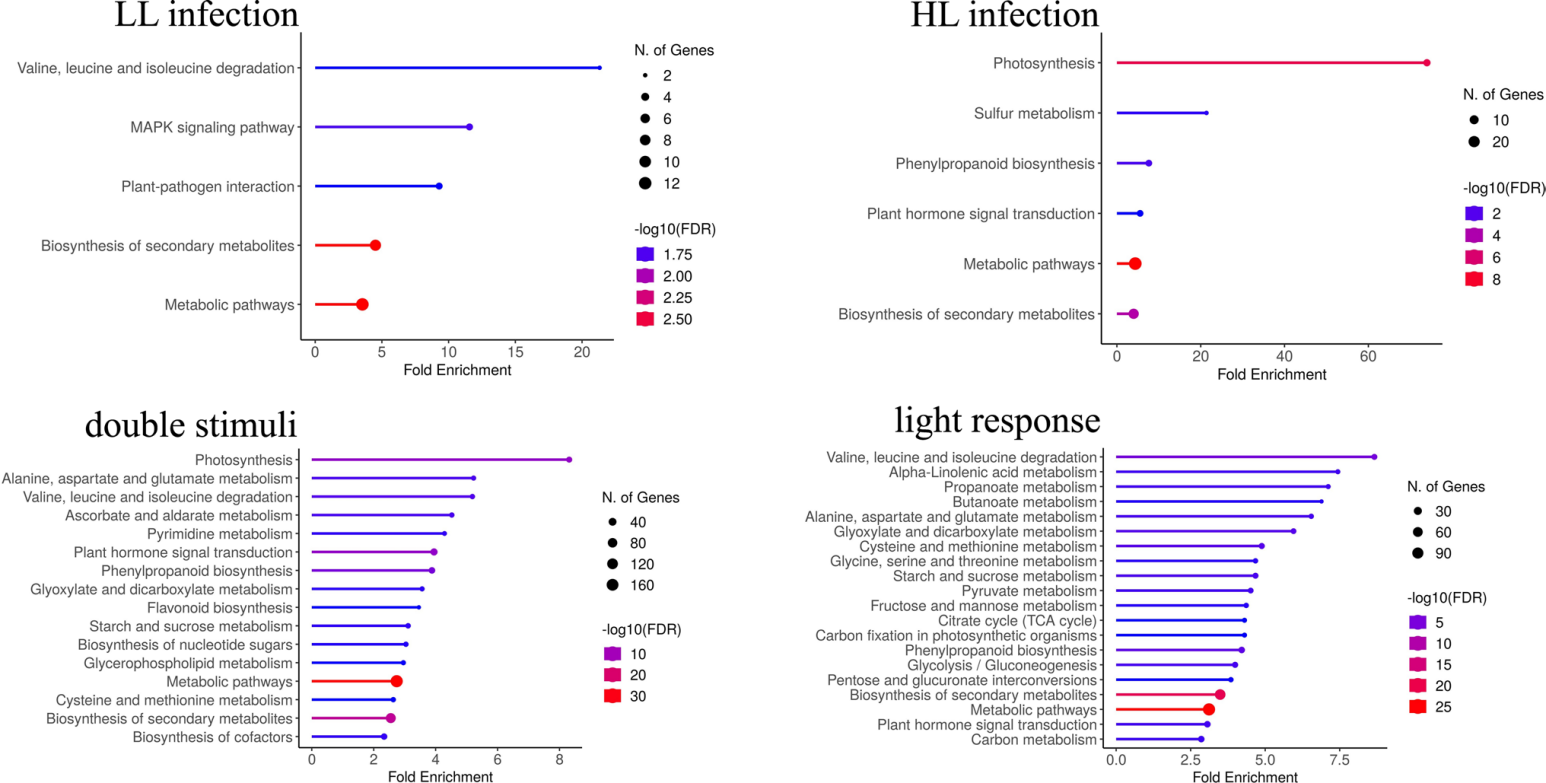
Gene ontology enrichment analysis within DEG groups. The KEGG pathways enriched in tomato roots due to *G. rostochiensis* parasitism under different light intensities

Screening large datasets of DEGs encounters problems with drawing more general conclusions; therefore, we employed ShinyGO v.0.77 software for functional categorization of DEGs and gene ontology (GO) enrichment analysis. Genes were classified according to four groups of GO terms: KEGG pathways, biological process (BP), molecular function (MF), and cellular component (CC) (see Fig. 6 and Supplementary Table 4). This approach allowed us to dissect processes being preferentially targeted upon potato cyst nematode parasitism in combination with environmental stimuli.

In all analyzed DEG groups, the most general category, “Metabolic Pathways” and also quite capacious “Biosynthesis of Secondary Metabolites” were consistently enriched. Specifically, among the LL-inf DEGs, notable enrichment was observed in “Valine, Leucine, and Isoleucine Degradation” as well as the “MAPK Signaling Pathway” categories (when KEGG pathways define the category) (see Fig. 6). Among other categories, the highest enrichment was evident in “Glutamine Family Amino Acid Catabolic Process” and”Negative Regulation of Hydrolase Activity” categorized according to BP, and in “Fatty Acid Binding” and “Water Channel Activity” when MF determines functional category (refer to Supplementary Table 4). As expected, the HL-inf DEGs were enriched in pathways associated with “Photosynthesis.” Moreover, two additional categories, “Sulfur Metabolism” and “Phenylpropanoid Biosynthesis,” were highly enriched (see Fig. 6). These findings were consistently supported across both GO terms for BP and MF (refer to Supplementary Table 4). The most pronounced KEGG pathway overrepresentation among the double stimuli DEGs was “Photosynthesis” followed by “Alanine, Aspartate, and Glutamate Metabolism.” Notably, the “Valine, Leucine, and Isoleucine Degradation” pathway also showed enrichment among double stimuli DEGs, highlighting the significance of amino acid metabolism in plants exposed to a complex environment (see Fig. 6). Additionally, “Gamma-Aminobutyric Acid Metabolic Process” emerged as a highly enriched category for BP, while “Cytidine Triphosphate (CTP) Synthase Activity” stood out for MF. Moreover, in both category groups — BP and MF — we found expected functional enrichments related to plant-nematode interaction, such as stress response, phytohormone regulation, defense response, cell wall remodeling, and ROS signaling.

The association of a gene product with a gene ontology term does not always proportionally reflect its engagement in a given molecular function, cellular component, or biological process. Therefore, dissecting domains from complex multidomain arrangements and conducting enrichment analysis could provide valuable supplementary insights needed for understanding large candidate lists. We found the Analysis of Motif Enrichment (AME; McLeay and Bailey 2010) particularly helpful in interpreting our DEGs lists (refer to Table 1). Among LL-inf DEGs, five significantly enriched motifs were identified. Among them, three were unique for LL-inf: “Hydroxymethylglutaryl-coenzyme A reductases signature 2” (PS00318), “cysteine-rich secretory proteins—CRISP family” (PS01009), and “nitrite and sulfite reductases iron-sulfur/siroheme-binding site” (PS00365). The Hydroxymethylglutaryl-coenzyme A (HMG-CoA) reductase is a key enzyme in the mevalonate pathway, responsible for biosynthesizing isoprenoids including sterols (Friesen and Rodwell 2004). Both LL-inf and HL-inf DEGs share the enriched motif named “Soybean trypsin inhibitor (Kunitz) protease inhibitors family” (PS00283). Three DEGs groups, the LL-inf, double stimuli, and light response, share the enriched signature “Zinc finger RING-type” (PS00518), describing a conserved RING domain pivotal in the ubiquitination pathway. The potential role of proteins with this motif in stress responses could be linked to modulating protein abundance or turnover via ubiquitin-mediated protein degradation (Sun et al. 2019). Among HL-inf DEGs, the “Cytochrome P450 cysteine heme–iron ligand signature” (PS00086) is enriched, while among the double stimuli DEGs, three significantly enriched motifs were found: “Eukaryotic and viral aspartyl proteases active site” (PS00141), “Serine/Threonine protein kinases active-site” (PS00108), and “2Fe-2S ferredoxin-type iron-sulfur binding region” (PS00197). These results were partially confirmed by a similar, recently published tool — Simple Enrichment Analysis (SEA; Bailey and Grant 2021; refer to Supplementary Table 5). The presence of the aforementioned motifs within protein sequences may be linked to particular condition-specific functions and regulatory roles in plant-biotic interactions.

**Table 1:** The regulatory protein motifs enriched in analyzed datasets. The Analysis of Motif Enrichment (AME) method was employed to identify over-represented motifs within the proteins encoded by DEGs from *G. rostochiensis*–attacked tomato roots under different light conditions

DEG group	Motif ID (prosite)	Motif alternative ID	Consensus	*p*-value	adj_*p*-value	*E*-value
LL-inf	PS00318	HMG_COA_REDUCTASE_2	LGXLGGGT	5.01e-4	2.50e-3	2.45e0
	PS01009	CRISP_1	GRFSALLWXXS	1.44e-3	2.89e-3	2.82e0
	PS00365	NIR_SIR	SGCXXXCXXXXXXELGL	1.44e-3	2.89e-3	2.82e0
	PS00283	SOYBEAN_KUNITZ	LXDXNGKXLXXXXXYXL	1.44e-3	2.89e-3	2.82e0
	PS00518	ZF_RING_1	CXHXLCXXCL	1.44e-3	4.33e-3	4.23e0
HL-inf	PS00283	SOYBEAN_KUNITZ	LXDXEGKXLXXXXXYXL	2.34e-4	7.03e-4	6.88e-1
	PS00086	CYTOCHROME_P450	FSXGXKXCLG	3.85e-3	7.69e-3	7.52e0
Double stimuli	PS00518	ZF_RING_1	CXHXLCXXCL	1.75e-6	2.27e-5	2.22e-2
	PS00141	ASP_PROTEASE	LLSDSGSSXSXL	1.48e-4	1.18e-3	1.16e0
	PS00108	PROTEIN_KINASE_ST	LXYXDLKXXNLLL	1.48e-4	1.48e-3	1.44e0
	PS00197	2FE2S_FER_1	CXXGXCSSC	1.48e-4	1.62e-3	1.59e0
Light response	PS00518	ZF_RING_1	CXHXLCXXCL	7.42e-6	9.64e-5	9.43e-2
	PS00198	4FE4S_FER_1	CXXCXXCXXXCG	9.56e-4	4.77e-3	4.67e0

### Consistency of RNA-seq results with other transcriptomic studies

**Fig. 7:**
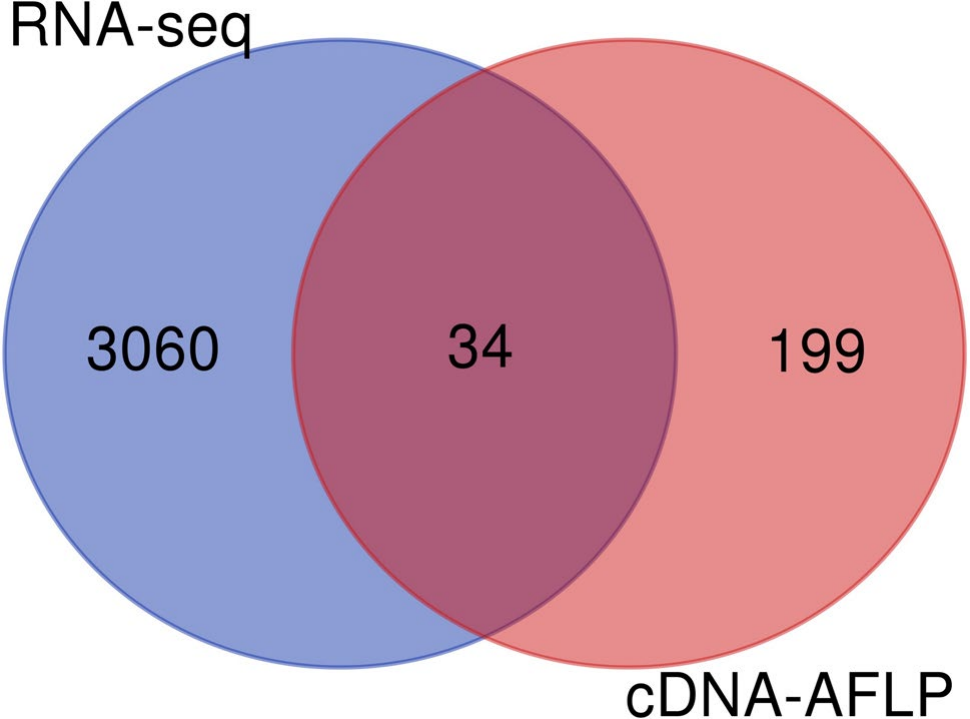
Comparison of tomato transcriptomic responses to *G. rostochiensis* parasitism. Venn diagrams showing the number of mapped DEGs described here and in cDNA-AFLP approaches

Meta-analyses of transcriptomic results obtained by different methods help identify strong candidates for further research. Despite our RNA-seq data reflecting gene expression changes in whole nematode-infected root systems 14 days post inoculation (since syncytia initiation was not synchronized, root samples contained 10–14-day-old feeding structures), we compared the DEG list to earlier studies on the same species, where cDNA-AFLP was used to monitor transcriptome changes at 1, 3, 7, and 14 dpi with dissected syncytia (Swiecicka et al. 2009; Święcicka et al. 2017). Thirty-four DEGs overlapped between these two approaches — 19 upregulated, 13 downregulated, and 2 were stable in the RNA-seq study (refer to Fig. 7; Supplementary Table 6). Notably, 65% of the DEGs found in both analyses (22 genes) confirmed expression trends (up- or downregulation upon nematode infection at any time point), while 35% (12 DEGs) demonstrated an inconsistent expression pattern. Among transcripts with a consistent expression pattern, there are several intriguing candidates for future research. The highly upregulated gene, *Solyc11g021060.2*, encodes the TOMARPIX proteinase inhibitor (with a 3.09 log2FC in the double stress comparison). Conversely, a strongly downregulated gene observed in the double stress response was *Solyc08g014130.3*, which encodes Isopropylmalate synthase (with a − 1.44 log2FC).

## Discussion

The divergence in plant responses to the co-occurrence of abiotic and biotic stresses is well known. The variability in these responses is influenced by factors such as the magnitude and duration of the applied stress, the specific plant species involved, and the developmental stage of the plant (Zandalinas and Mittler 2022; Georgieva and Vassileva 2023). Examining the plant’s response to a combination of factors involves an assessment of whether a physiological and molecular response is induced. In our study, we identified genes that exhibit differential expression in response to the combination of simultaneous action of environmental stimuli in tomato plants — nematode pest attacking roots and higher light intensity applied to leaves.

### The impact of elevated light intensities on tomato seedlings

Plants depend on light for their survival, yet excessive light can have detrimental effects. Beyond a critical threshold, high light (HL) intensity not only directly impairs the photosynthetic apparatus but also induces oxidative stress, leading to photodamage and photoinhibition. Moreover, elevated light levels trigger the generation of ROS, increasing the risk of widespread cellular damage (Li et al. 2009; Roeber et al. 2021). To monitor the physiological state of tomato seedlings, we measured chlorophyll a fluorescence. To induce a HL response, we transferred plants from low light intensities (50–60 μmol m^−2^ s^−1^) to HL conditions (350–400 μmol m^−2^ s^−1^). Such levels of light intensities are not unusual in natural environment. However, the seven-fold increase in intensity was expected to modify PSII efficiency (Dietz 2015). Here, only two parameters demonstrated statistically significant increases 1 and 3 dpt: PSII quantum yield (Fig. 1C) and photochemical fluorescence quenching (Fig. 1E). HL stress typically leads to a loss of photosynthesis efficiency, represented by a decrease in the *F*_v_/*F*_m_ ratio (Baker 2008). Therefore, we may conclude that our HL conditions were relatively mild. However, it is noteworthy that we avoided a temperature shift of the root system, which is often an overlooked variable in such experiments. Several reports on tomato plants grown under similar light intensities yield inconsistent results. Takagi and colleagues (2019) demonstrated that plants cultivated or transferred to HL intensities exhibited enhanced efficiency of photosystem II (PSII), whereas a study conducted by Pascual and colleagues (2023) found that the application of light intensities around 700 μmol m^−2^ s^−1^ for a duration of 9 h resulted in a reduction of PSII efficiency and photosynthetic rate in tomato plants.

Even at applied HL intensities, the molecular response was evident, showing the activation of pathways connected with much stronger abiotic or biotic stimuli (Fig. 2). During the first days after transfer to HL, we observed upregulation of typical markers of defense and antioxidant pathways, while genes that may conserve energy and reduce susceptibility to light-induced stress were downregulated. Surprisingly, we found downregulation of the *ELONGATED HYPOCOTYL5* (*HY5*) transcription factor, which induction may be a marker of stress response. HY5 is involved in systemic shoot–root signaling in response to light stress as well as in maintaining homeostasis of carbon and nitrogen metabolism under ambient light conditions (Chen et al. 2016). Many of the observed molecular effects were temporal and disappeared after 3 dpt, indicating effective adaptation. However, even short-term response activation may interfere with a particular phase of nematode parasitism, such as the migration phase, syncytium establishment and functioning, which engage distinct pathways (Siddique et al. 2022; Matuszkiewicz and Sobczak 2024). It is worth noting that shaded roots appear to be more sensitive than illuminated shoots and showed a more complex reaction. Most plant nematode studies overlook this aspect, whereas it is known that direct illumination of roots cultivated in vitro alters their morphology, cellular, biochemical, and molecular responses (Cabrera et al. 2022). Moreover, reducing gas exchange is an additional modifying factor for root growth phenotype (Matuszkiewicz et al. 2019). To minimize the influence of such factors, we routinely use root shading and air-permeable Petri plate sealing instead of Parafilm.

### Light-mediated modulation of root response to* G. rostochiensis*

The core premise of this study hinges on the understanding that exploring specific aspects of plant interactions with the environment or other organisms in laboratory conditions often yields results that inadequately reflect the phenomena observed in natural settings. Consequently, the anticipation of light-mediated modifications in root responses following nematode attacks was justified, yet the extent and the specific pathways involved remain to be fully elucidated (Pandey et al. 2015; Zandalinas and Mittler 2022). The decrease in PSII yield observed in infected tomato plants cultivated in LL conditions aligns with expectations. However, infected plants transferred to higher light intensities exhibited a PSII yield comparable to that of control plants. Overall, it can be inferred that infected plants under LL conditions manifested a photoinhibition-like response (Fig. 3). This observation is consistent with findings by Schmitz et al. (2006), who demonstrated similar fluorescence parameter alterations in sugar beet plants infected with beet cyst nematodes (*H. schachtii*) under moderate light intensities (200 μmol m^−2^ s^−1^). During the early stages of beet infection by *H. schachtii*, reductions in photosynthetic efficiency occur, followed by declines in transpiration and photosynthetic processes. These declines are attributed to stomatal closure, impaired mineral nutrient uptake, and reductions in chlorophyll and nitrogen content in the leaves of infected plants. However, changes in photosynthetic efficiency in *H. schachtii*–infected *A. thaliana* appear slightly different (Labudda et al. 2018). In this case, only minor changes in photosynthetic efficiency occur, which is consistent with our results, including the stable level of tomato seedling susceptibility (Fig. 4). Interestingly, in our previous studies, we documented changes in the susceptibility of *A. thaliana* infected with *H. schachtii*, which were dependent on the aforementioned ventilation conditions of the Petri plates (Matuszkiewicz et al. 2019). Another factor that should be considered is the interplay between sugar- and light-induced signaling pathways and its effect on the condition of the photosynthetic apparatus. Most plant-nematode interaction experiments in vitro involve the supplementation of the medium with sucrose as an additional carbon source. It is generally assumed that plants in vitro are subjected to persistent low light, which is a limiting factor for photosynthesis and morphogenesis. Consequently, the supplementation of sucrose to the medium is often deemed necessary, influencing various signaling components, including receptors, kinases, transcription factors, and hormones (Gago et al. 2014). The crosstalk between these pathways may modify plant responses to environmental factors and modulate their growth and survival strategies under changing conditions (Ciereszko 2018). This highlights the importance of considering such multivariate analyses in interpreting experimental outcomes.

### Tomato transcriptomic reprogramming under combined environmental stimuli

Transcriptome profiling using RNA-seq has become a pivotal approach for diagnosing plant responses to stress. Our investigation encompassed nematode-infected and uninfected tomato roots grown persistently under both low and high light conditions, as well as those transferred from low to higher light intensities. Despite the stable susceptibility of tested tomato cultivar to *G. rostochiensis* across varying light conditions, significant changes in the abundance of differentially expressed root transcripts were observed. Our findings underscore the substantial influence of light on gene expression. Furthermore, the synergistic interaction between light and nematode responses produced more DEGs than the sum of individual stimuli. Such light stimulation, crucial for activating defense/resistance responses, has also been observed in plant-pathogen interactions, particularly with bacterial pathogens (Trotta et al. 2014). Another example of this mechanism was described by Gao et al. (2020), who demonstrated that nucleotide-binding NLR Rpi-vnt1.1 proteins require light for conferring resistance against *Phytophthora infestans* races, specifically those releasing the effector protein AVRvnt1.

Analyzing the DEGs with representation enrichment tools across all generated RNA-seq data, we consistently found two KEGG categories overrepresented, namely “Metabolic Pathways” and “Biosynthesis of Secondary Metabolites” which serve as nonspecific markers of environmental factor response (Fig. 6). However, among the DEGs, there were some more specific enrichments, such as “Valine, Leucine, and Isoleucine Degradation” and the “MAPK Signaling Pathway” in the LL-inf group. The degradation of branched-chain amino acids (BCAAs) is connected with energy production and nitrogen recycling and may be part of the classical growth-defense trade-off (Hildebrandt et al. 2015; He et al. 2022). The MAPK signaling pathway is also typical in plant-pathogen interactions, involved in signal transduction to maintain ROS production and integrate signals from JA and SA pathways (Taj et al. 2010).

New categories emerged in the group of DEGs after nematode infection of plants grown under HL conditions, namely “Photosynthesis,” “Sulfur Metabolism,” and “Phenylpropanoid Biosynthesis.” The double stimuli DEGs were overrepresented by “Photosynthesis,” “Alanine, Aspartate, and Glutamate Metabolism,” and another amino acid-related pathway “Valine, Leucine, and Isoleucine Degradation.” These pathways emphasize the importance of amino acid metabolism in plants exposed to complex environmental stimuli, including moderate parasite pressure and extensive light intensity variation. Nematode infection evokes multidirectional changes in roots involving amino acids as substrates for hormones and newly synthesized proteins needed for developmental reprogramming during syncytium formation and feeding parasitic nematodes. The observed synergy between the responses triggered by light exposure and nematode infection indicates substantial enrichment in processes essential for plant adaptation to stress conditions, such as energy allocation, phytohormone crosstalk, and enhanced secondary metabolite production. The integration of these responses is likely context-dependent, specific to environmental stimuli, the type of plant-pathogen interaction, and their intensity.

Gene ontology analysis was supplemented with motif enrichment approaches (McLeay and Bailey 2010). For example, the “cysteine-rich secretory proteins—CRISP family” motif was enriched in the LL DEG group. CRISPs belong to a family of proteins with conserved cysteine residue arrangements present in animals and involved in gamete interaction (Gonzalez et al. 2021). In plants, proteins with that motif could be found among pathogenesis-related proteins (Han et al. 2023). Another pathogenesis-related and enriched motif was found in LL-inf and HL-inf DEGs — “Soybean trypsin inhibitor (Kunitz) protease inhibitors family,” typically occurring in protease inhibitors commonly linked to defense responses. Interestingly, we also observed enriched signatures of antagonistic activities, such as “Eukaryotic and viral aspartyl proteases active site,” typical for proteolytic enzymes involved in stress-related processes such as protein degradation, plant senescence, and programmed cell death (Simões and Faro 2004).

The above-listed statistically significant molecular characteristics are complex and difficult to summarize. We may speculate that in compatible plant-nematode interactions, an additional environmental factor (e.g., higher light intensities) can both enhance and inhibit specific plant defense responses. In the face of such antagonistic responses, other pathways are activated, which on a molecular level resemble the priming phenomenon (Nair et al. 2022). Whether resulting stress tolerance was enhanced requires further studies with higher parasite inocula, more HL levels, and different stressors.

## Conclusion

Stress factors in nature typically do not occur in isolation. The combination of nematode infection in roots and significant variations in light intensity on leaves induces a greater local and systemic response than the sum of the responses to each stressor individually. Our model revealed an increasing role of amino acid metabolism and hormonal regulation. Conversely, processes traditionally classified as “plant-pathogen related” appear to exhibit diminished relevance in the context of complex environmental factors. Therefore, research on genes implicated in the response to stress combinations is crucial for comprehending the molecular pathways associated with such responses and for the development of more resilient cultivars. This research is particularly vital in the face of climate changes, where plants will likely face multiple, simultaneous stressors, making the development of multi-stress resistant crops a priority for sustainable agriculture.

## Supplementary Information

Below is the link to the electronic supplementary material.

Supplementary file1 (PDF 226 KB)

Supplementary file2 (XLSX 16 KB)

Supplementary file3 (XLSX 10 KB)

Supplementary file4 (XLSX 610 KB)

Supplementary file5 (XLSX 56 KB)

Supplementary file6 (XLSX 18 KB)

Supplementary file7 (XLSX 102 KB)

## Data Availability

The raw data supporting the conclusions of this article will be made available by the authors, without undue reservation.
