# Supplementary material for: Identification of genes involved in the tomato root response to Globodera rostochiensis parasitism under varied light conditions
Source: J Appl Genet. 2024 Aug 14;66(1):47–61. doi: 10.1007/s13353-024-00897-6 (PMC11762221; doi:10.1007/s13353-024-00897-6)
Supplement: Supplementary file 1 — Supplementary file1 (PDF 226 KB) [file 13353_2024_897_MOESM1_ESM.pdf]

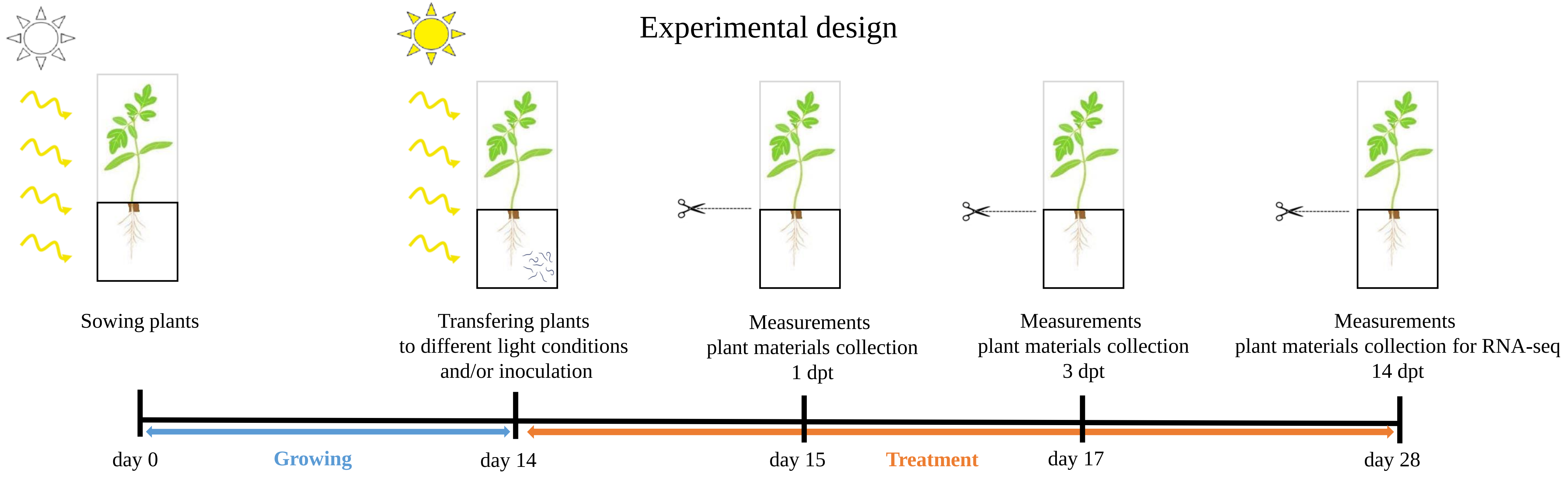

Tested combinations:
LL – plants were grown for the whole time in low light conditons
LLtoHL – plants after 2 weeks of growth in low light conditions were transfered to higher light intensities
HL – plants were grown for the whole time in high light conditon
inf – infected plants

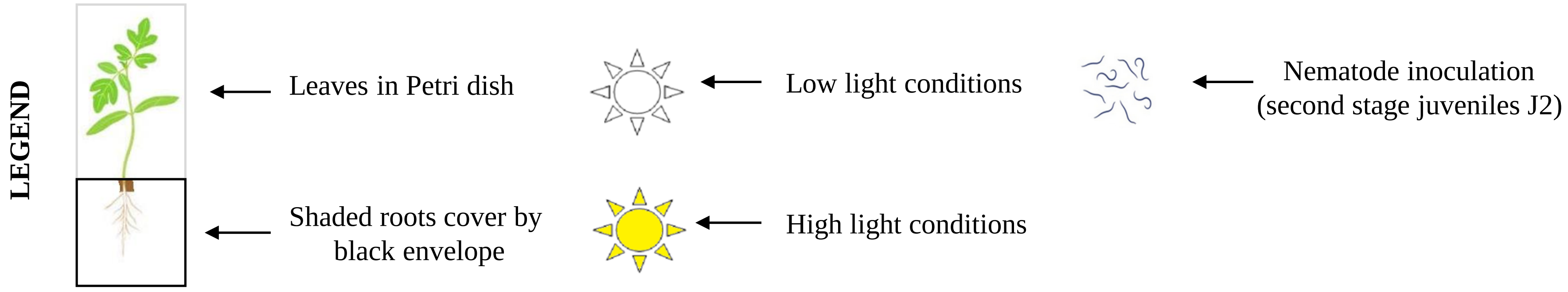

Supplementary Figure 1 – Experimental design
